# Light-guided spectral sculpting in chiral azobenzene-doped cholesteric liquid crystals for reconfigurable narrowband unpolarized light sources

**DOI:** 10.1515/nanoph-2025-0455

**Published:** 2025-12-04

**Authors:** Pravinraj Selvaraj, Ming-Hong Yuan, Cheng-Kai Liu, Ko-Ting Cheng

**Affiliations:** Department of Optics and Photonics, 529843 National Central University, Taoyuan 320317, Taiwan

**Keywords:** cholesteric liquid crystals, broadband reflection, narrowband spectral filtering, light-responsive phenomena, molecular photo-switches

## Abstract

Precise manipulation of Bragg reflection in cholesteric liquid crystals (CLCs) is essential for advancing reconfigurable optics. However, existing photo-responsive material-doped CLC technologies that rely on single-wavelength photoisomerization encounter several challenges, including slow response times, limited tunability, inadequate spatial control, and instability caused by pitch variations due to diffusion. Here, we present a robust dual-wavelength photoisomerization method to simultaneously achieve *trans*-to-*cis* and *cis*-to-*trans* photoisomerization of chiral azobenzene-doped CLCs, which enables broadband, reversible, and spatially addressable control over the Bragg reflection spectrum. By employing counterpropagating laser beams at 405 nm and 532 nm, we precisely control the *trans*–*cis* isomerization dynamics of azobenzene chiral dopants, achieving spectral shifts exceeding 100 nm primarily through reversible modulation of the helical pitch of the CLCs. Furthermore, manipulating the intensity ratio and geometry of the excitation beams allows for tailored pitch gradients, reflection bandwidths, and central wavelengths with remarkable fidelity. Our approach enhances pitch boundaries and reduces molecular diffusion, facilitating the micrometer-scale patterning of optical textures, which surpasses traditional single-wavelength methods. Additionally, we present an innovative narrowband spectral filtering technique by sequentially transmitting light through pitch-selective CLC regions under circular polarization control. This reconfigurable manipulation strategy paves the way for developing programmable photonic systems, including adaptive optics, diffractive optics, and tunable displays.

## 1 Introduction

Nature showcases extraordinary structural coloration, particularly through the complex nanoscale architectures in butterfly wings [1] and beetle exoskeletons [2]. These biologically inspired structures facilitate dynamic color changes essential for functions such as camouflage [3], [4] and visual display [5]. Consequently, researchers have sought to harness responsive soft materials to develop self-assembled photonic structures. Cholesteric liquid crystals (CLCs) are particularly noteworthy for their ability to form helical superstructures [6], [7], [8], [9] that produce a photonic bandgap (PBG), selectively reflecting circularly polarized light in accordance with Bragg’s law [10]. The unique optical properties of CLCs have led to diverse applications, including display technologies [11], diffraction gratings [12], imaging systems [13], and smart window applications [7], [14], [15]. However, achieving precise and reversible control over the helical superstructures and their optical characteristics in response to external stimuli remains a challenging endeavor in materials engineering.

A promising strategy to address these challenges involves integrating chiral photo-switches, which enable the dynamic manipulation of molecular arrangements and optical properties in response to light stimuli [2]. Over the past decade, various chiral molecules have been developed as dopants within CLC systems. Among these, chiral photo-switches such as azobenzenes [16], [17], [18], molecular motors [19], [20], [21], and diarylethenes [22], [23] have garnered significant attention due to their ability to reversibly alter the molecular organization of LCs in response to light stimuli. This responsiveness facilitates tunable Bragg reflections, thereby supporting the development of reconfigurable photonic devices. Traditional methodologies have primarily focused on blending chiral dopants with photo-switchable molecules; however, challenges persist in achieving uniform and thermally stable tuning of the helical structure. Notably, diarylethenes, which consist of two thiophene units connected by an ethene bridge, are particularly esteemed for their impressive fatigue resistance, reversibility, and thermal stability [24], [25], [26]. Incorporating chiral side groups, such as binaphthyl moieties, onto achiral diarylethenes can produce robust and thermally stable chiral dopants [27]. Nevertheless, the minimal conformational changes occurring during the photocyclization process often fail to effectively influence the pendant chiral side groups within CLCs, resulting in limited helicity variations and complications in achieving helical inversion. Furthermore, these extrinsic chiral modifications frequently yield low asymmetric induction during photocyclization, producing diastereomers with opposing stereogenic centers [24]. Consequently, this phenomenon can lead to the emergence of multiple helical domains, contributing to orientation disorder within the LC matrix and ultimately diminishing optical efficiency.

Azobenzene-based chiral dopants have gained attention for their ability to undergo reversible *trans*–*cis* photoisomerization, facilitating light-driven modulation of helical twisting power (HTP) [24], [28], [29]. However, much of the current research emphasizes single-wavelength or broadband exposure without selective control, which restricts precise spectral tunability and may lead to challenges such as uncontrolled isomerization and uneven pitch distributions, resulting in limited spectral selectivity and inefficient suppression of off-band transmission due to insufficient modulation depth and static structural configurations. These constraints pose significant challenges for practical applications in narrowband light filtering, where precise control over wavelength and polarization is crucial. To overcome these challenges, previous studies have examined using azobenzene-doped CLCs for photonic applications, highlighting the advantages of dual-wavelength exposure. For instance, Chen et al. developed a quantum dot-embedded CLC laser incorporating a chiral azobenzene moiety, enabling reversible PBG tuning and lasing wavelength tuning through alternating dual-wavelength (UV and blue light) exposure [30]. Although this method demonstrated optical stability and tunability, the tuning range remained limited to ∼60 nm for the PBG and 40 nm for the lasing wavelength. Moreover, it did not provide precise spatial control over pitch gradients and bandwidth, which is critical for advanced photonic devices. Similarly, Fuh et al. introduced an optical filter employing two phototunable CLC cells in a reflection mode, facilitating adjustment of the central wavelength from 510 nm to 628 nm, with bandwidths varying from 13 nm to 79 nm through dual-beam exposure [31]. However, this configuration primarily addressed wavelength and bandwidth tunability while neglecting spatial pitch modulation and dynamic reconfigurability of the reflection band, thereby constraining its applicability in designs necessitating meticulous spectral control. Additionally, White et al. demonstrated photo-induced broadening of CLC reflectors through UV exposure, significantly enhancing the reflection bandwidth from approximately 100 nm to as much as 1700 nm [32]. Despite this considerable enhancement, the method relied on passive exposure and did not incorporate reversible or spatially controlled modulation. Furthermore, Wu et al. reported on photoisomerization-driven molecular migration in azobenzene polymer systems, highlighting the formation of UV-assisted surface relief gratings through light-induced mass transport [33]. However, this technique is constrained by its reliance on single-wavelength photoisomerization, which limits bidirectional, reversible control over helical pitch and Bragg reflection spectra. Such limitations hinder the dynamic and reconfigurable manipulation of photonic properties, which are essential for advancing adaptive optical devices.

**Figure 1:**
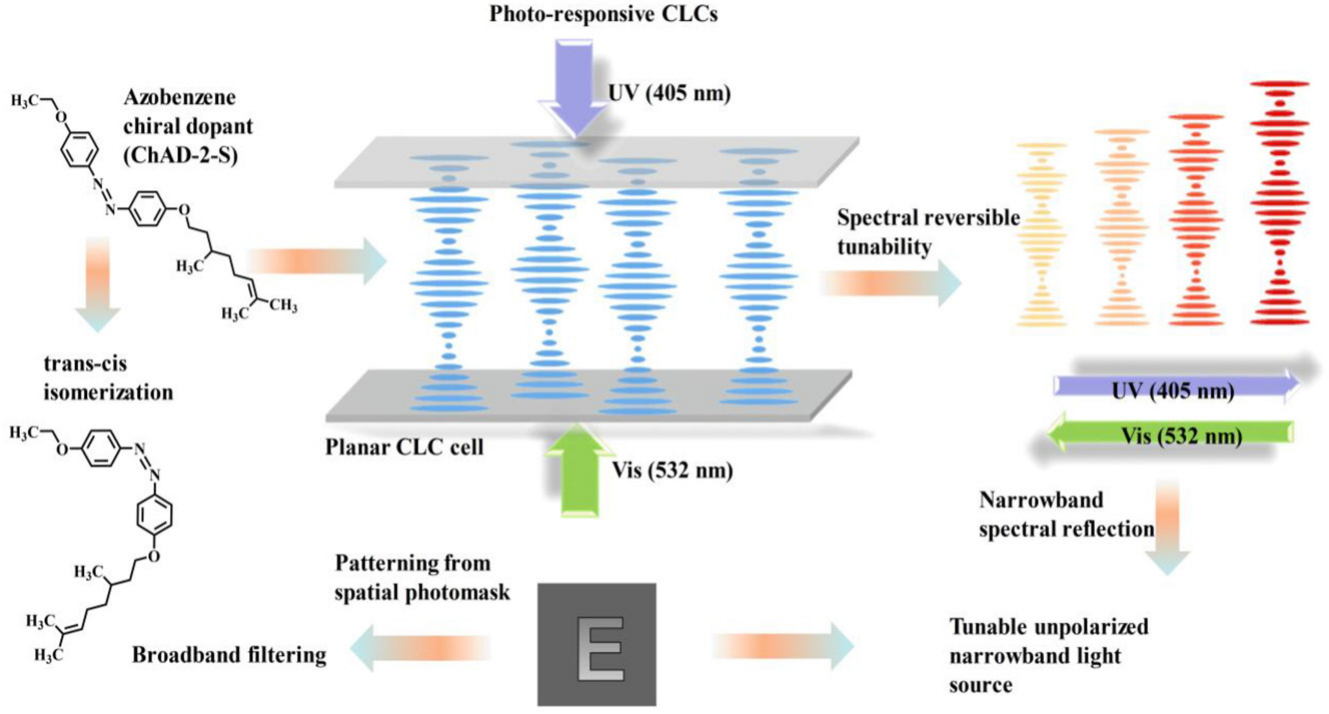
A schematic illustration of the dual-wavelength exposure on the *azo*-*n*CLC cell and its promising applications in spectral sculpting.

To tackle these challenges, we propose a spectrally reconfigurable chiral azobenzene-doped negative CLC (*azo*-*n*CLC) device, which enables dynamic and reversible control of the PBG via dual-wavelength photoisomerization using UV (405 nm) and green (532 nm) light. This innovative device enables the simultaneous *trans*–*cis* and *cis*–*trans* isomerization of chiral azobenzene dopants under specific optical stimuli (Figure 1). Unlike conventional single-wavelength techniques, our device employs counterpropagating laser beams at UV and green wavelengths to achieve precise control over the isomeric state of chiral azobenzene-based dopants. This dual mode activation exploits distinct π–π* transitions induced by UV light and n–π* transitions stimulated by visible light, thereby promoting efficient and reversible photoisomerization. The resulting transformation alters molecular geometry and dipole moment, thereby adjusting the HTP of the chiral dopants and dynamically modulating the supramolecular pitch of the CLC. Consequently, the CLC cell exhibits a broadband and tunable reflection from the visible to the near-infrared spectrum. When encapsulated within a planar-aligned cell, the CLC forms a helical superstructure that selectively reflects circularly polarized light according to Bragg’s law. Moreover, we showcase spatially selective photopatterning within the CLC device. By employing a photomask patterned in the shape of the letter “E” in the path of the 405 nm UV laser. This approach enables localized *trans*–*cis* photoisomerization of azobenzene dopants. This method generates spatial variations in helical pitch, resulting in a patterned modulation of the photonic bandgap across the film. We achieve pitch gradients and spectral shifts with submicron spatial resolution by incorporating left- and right-handed chiral azobenzene dopants within a nematic matrix. Notably, including bulky tail groups mitigates excessive diffusion, allowing for sharper pitch gradients and enhanced surface anchoring, both crucial for obtaining spatially resolved spectral features [34], [35], [36]. This approach facilitates precise, reversible manipulation of the reflection band’s position and bandwidth, while offering greater adaptability for diverse photonic applications. By effectively overcoming previous limitations, such as poor reversibility, restricted modulation depth, and uncontrolled diffusion, our methodology paves the way for advancements in adaptive optics, tunable filters, wavelength-selective mirrors, and high-resolution reflective displays in next-generation photonic technologies.

## 2 Results and discussion

### 2.1 Tunable reflection in azo-nCLCs through dual-wavelength azobenzene photoisomerization

**Figure 2:**
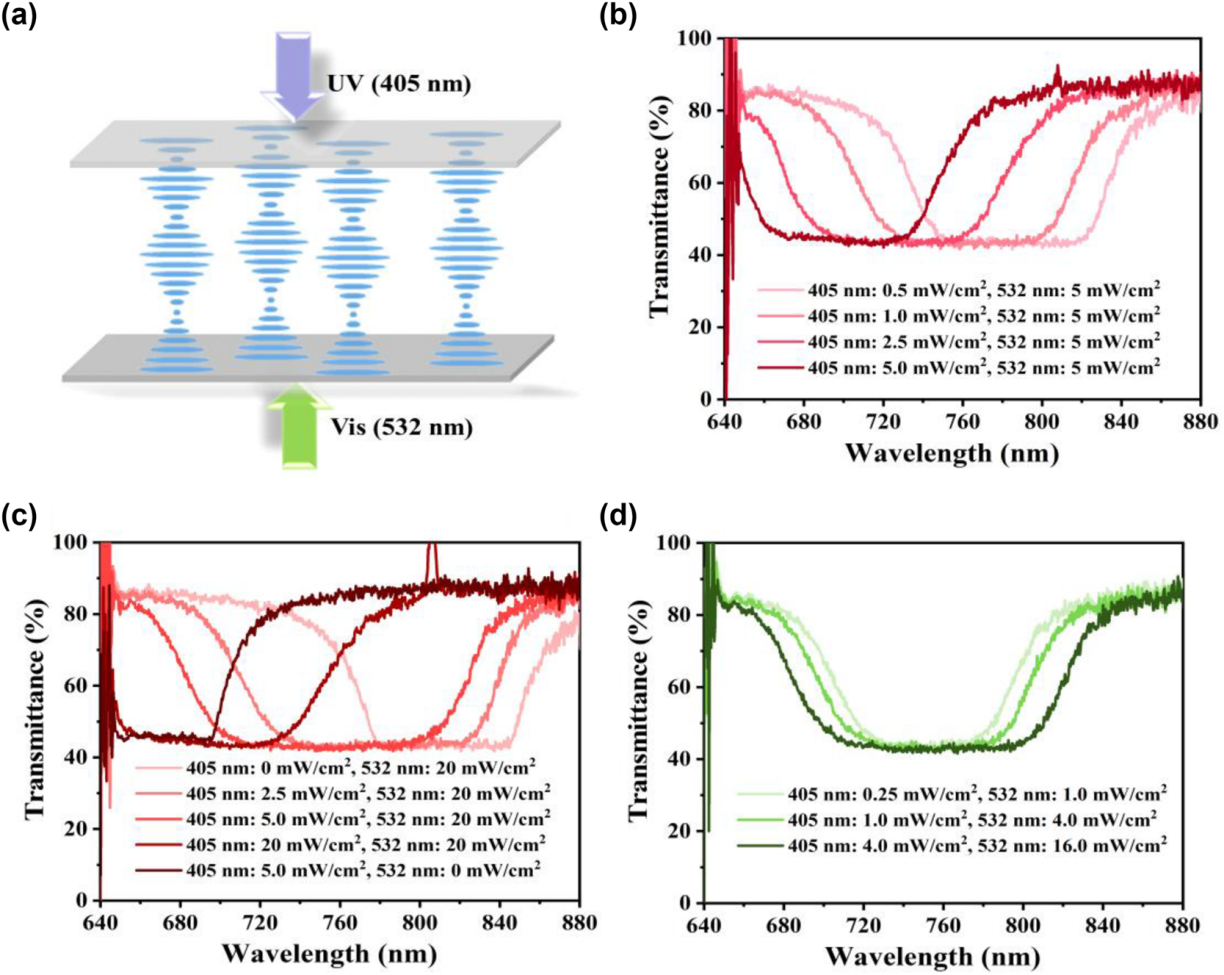
Spectral modulation of the A*zo-n*CLC cell under dual-wavelength exposure. (a) Schematic illustration of the *azo*-*n*CLC cell under simultaneous dual-wavelength counterpropagating beam exposure. (b) Transmission spectra obtained from continuous dual-beam exposure with a fixed 532 nm laser at 5 mW/cm^2^ and varying intensities of a 405 nm laser. (c) Transmission spectra recorded with a fixed 532 nm laser at 20 mW/cm^2^, varying the intensity of the 405 nm laser. (d) Simultaneous transmission spectra of the cell exposed to varying intensities of 405 nm and 532 nm lasers, maintaining proportional intensity relationships. During the spectra measurements shown in (b)–(d), the *azo*-*n*CLC cell was continuously applied an AC voltage of 100 *V*_pp_ with a frequency of 1 kHz.

To elucidate the spectral tunability of photo-switchable *azo-nCLC* cells, we examined the effects of counterpropagating 405 nm and 532 nm laser beams (Figure 2(a)). The pristine CLC cell exhibited a right-handed helical structure, displaying a near-infrared Bragg reflection centered at 811 nm (bandwidth: 73 nm), as determined by the interplay between the left-handed azobenzene chiral dopant (ChAD-2-S) and the right-handed dopant (Table S1, Figure S1). Upon exposure to 405 nm light, *trans*–*cis* photoisomerization of the azobenzene units through π–π* excitation results in a 180° rotation around the N=N bond. The resulting bent conformation of the *cis*-isomer leads to a reduced HTP, causing a contraction of the cholesteric pitch and a subsequent blue shift of the reflection band to 665 nm (bandwidth: 60 nm), as shown in Figure S1. Subsequent exposure to 532 nm light promotes *cis*–*trans* back-isomerization via n–π* excitation and nonradiative decay, restoring the original higher HTP and causing a red shift in the reflection band. The orientation kinetics of the azobenzene dopants reveal rapid molecular reorientation during both *trans–cis* and *cis–trans* isomerizations. The bent *cis*-isomers disrupt local director alignment, leading to a decrease in effective HTP, while back-isomerization reinstates molecular order. This reversible molecular reorientation is crucial for the observed dynamic and stable spectral tuning. This bidirectional spectral tuning primarily derives from the photo-controlled variations in helical pitch induced by the *trans–cis* isomerization of azobenzene molecules, while the average refractive index remains effectively unchanged. This reversible isomerization process facilitates dynamic and tunable spectral modulation. The observed spectral modulation characteristics align with theoretical predictions derived using the transformed-matrix method for anisotropic helical structures [37]. This method provides an exact formulation for the reflection spectra of CLCs, dependent on pitch and birefringence parameters, thereby enhancing the quantitative analysis of experimentally obtained dual-wavelength reflection-tuning results. Furthermore, no significant surface degradation, delamination, or photobleaching was observed during irradiation. The optical properties of the *azo-n*CLC samples remained stable across multiple cycles of dual-wavelength photoisomerization, demonstrating the exceptional photostability of this material system under the prescribed illumination conditions.

To further elucidate the optical properties of the photo-responsive LC superstructure, we implemented a dual-wavelength counterpropagating beam configuration, applying an AC field of 100 *V*_pp_ at 1 kHz across the *azo-n*CLC cell (Figure 2(b)). This external electric stimulus was crucial for establishing uniform planar alignment and minimizing scattering effects caused by azobenzene diffusion. Under the dual-wavelength exposure setup (Figure S2), a constant green light intensity of 5.0 mW/cm^2^ combined with varying UV intensities (0.5–5.0 mW/cm^2^) resulted in a progressive blue shift of the reflection peak from 785 to 698 nm, alongside a narrowing of the bandwidth from 81 to 73 nm (Table S2). This shift is attributed to the increased concentration of *cis*-isomers near the UV-illuminated surface, which induces a spatial pitch gradient along the helical axis. Regions adjacent to the green interface maintain a longer pitch owing to *trans*-isomer predominance, while the resultant pitch gradient enhances spectral broadening. To further analyze the effects of optical intensity, we fixed the green light intensity at 20 mW/cm^2^ while varying the UV intensity (Figure 2(c)). At a UV intensity of 2.5 mW/cm^2^, the reflection bandwidth expanded to 101 nm, centered at 778 nm. Increasing the UV intensity to 5.0 mW/cm^2^ shifted the center to 754 nm and broadened the band to 109 nm. When both wavelengths were set to equal intensities of 20 mW/cm^2^, the center wavelength shifted to 694 nm with a narrowed bandwidth of 83 nm (Table S3). These results confirm that enhanced optical excitation intensifies the pitch gradient by elevating *cis*-isomers’ population near the UV-exposed surface, while the region illuminated by green light predominantly remains in the *trans* state. A comparative analysis of Tables (S2-S3) reveals that the bandwidth decreases at the spectral extremes (*λ*_e_ ≈ 840 nm and *λ*_o_ ≈ 690 nm) but reaches a maximum around intermediate wavelengths (∼750 nm). This behavior can be attributed to the relative distributions of *cis* and *trans* isomers: at spectral extremes, the dominance of a single isomer results in a weak pitch gradient and narrower bandwidth. Conversely, near 750 nm, the coexistence of both isomers produces the steepest gradient, yielding the broadest reflection band.

#### 2.1.1 Central wavelength and bandwidth control in dual-wavelength photoisomerization

In the realm of photonic applications, the ability to manipulate the central wavelength and bandwidth of the PBG through dual-wavelength photoisomerization offers a substantial advancement for dynamic photonic devices. The prepared *azo*-*n*CLC cell demonstrates independent bandwidth control without altering the central reflection wavelength by maintaining a fixed intensity ratio of UV to green light while adjusting the absolute power (Figure 2(d)). With a ratio of 1:4 (4.0/16.0 mW/cm^2^), the reflection was centered at 755 nm, featuring a bandwidth of 101 nm (Table 1). Reducing both intensities proportionately results in 75 nm and 62 nm bandwidths at central wavelengths of 753 nm and 751 nm, respectively. This indicates increased intensity leads to bandwidth broadening via enhanced pitch gradients, provided the intensity ratio remains constant. Moreover, varying the intensity ratios (20:1 and 1:1) produces interesting effects, as shown in Figure S3. At an intensity ratio of 20:1 (0.25/5.0 and 1.0/20.0 mW/cm^2^), the central wavelength (∼797 nm) remains stable while the bandwidth expands from 78 nm to 87 nm. Conversely, at an intensity ratio of 1:1 (5.0/5.0 and 20.0/20.0 mW/cm^2^), a minor shift in peak occurs from 698 nm to 694 nm, accompanied by an increase in bandwidth from 73 nm to 82 nm (Table S4). These findings demonstrate that enhancing overall intensity broadens bandwidth and preserves a stable intensity ratio, thereby strengthening control over the central wavelength. Furthermore, a comparative analysis of counterpropagating and copropagating beam geometries (Figures S4-S7) uncovers a noteworthy shift in reflection, with the *trans*-dominant configuration yielding 815 nm and a bandwidth of 78 nm, while the *cis*-dominant configuration reveals 666 nm and a bandwidth of 63 nm (Table S1-S3). Copropagating beams exhibit mutual attenuation, which confines isomerization gradients to the near-surface region, thus limiting spectral modulation. In contrast, counterpropagating beams facilitate a broader photo-induced *cis*–*trans* isomer distribution and enhanced pitch gradients, enabling more effective tuning of the optical response. Notably, even in the copropagating configuration (Figure S7, Table S3), an increase in intensity results in spectral broadening, albeit with reduced efficiency. The reflection shift demonstrates a nearly linear correlation with optical power at low intensities, transitioning to saturation at higher powers as photostationary equilibrium is established between the *trans* and *cis* isomers [38], [39]. A similar dependence of azobenzene orientation on light power has been observed in polymer-based azobenzene systems [40], where molecular reorientation correlates with excitation intensity. In contrast, our dual-wavelength *azo-n*CLC methodology facilitates concurrent control of both *trans–cis* and *cis–trans* isomerization, broadening the response to reversible helical pitch modulation and enhancing broadband spectral tunability. This research provides a solid foundation for advancing next-generation light-responsive photonic components, including dynamic optical filters, tunable mirrors, and adaptive light modulators.

**Table 1:** Center wavelengths ( $\bar{\lambda }$ ) and reflection bandwidths (Δ*λ*) of *azo*-*n*CLCs under dual-wavelength exposure with varying green and UV light intensities.

Light intensity (mW/cm^2^) 405 nm / 532 nm	$\bar{\lambda }$ (nm)	Δ*λ* (nm)
0.25 / 1.0	751	62
1.0 / 4.0	753	75
4.0 / 16.0	756	101

### 2.2 Enhanced boundary sharpness in CLCs through single and dual-wavelength exposure

**Figure 3:**
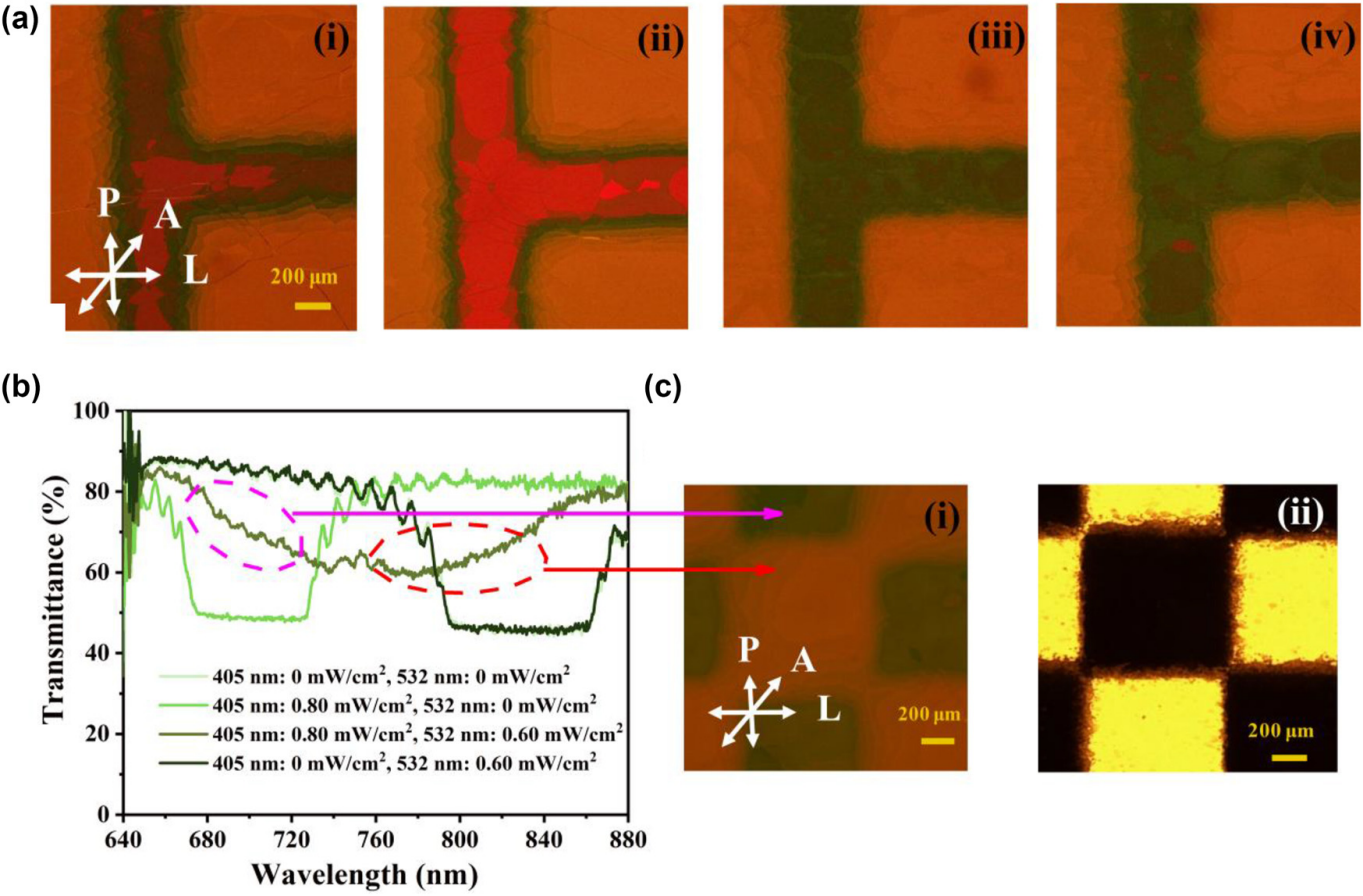
Optical response and switching dynamics of the A*zo-n*CLC cell under single- and dual-wavelength exposure. (a) POM images of binary CLCs fabricated under (i) single-wavelength 405 nm light exposure at 0.80 mW/cm^2^ for 5 min, (ii) 10 min, and (iii) dual-wavelength exposure combining 405 nm (0.80 mW/cm^2^) and 532 nm (0.60 mW/cm^2^) light for 5 min and (iv) 10 min. (b) Transmission spectra of binary CLCs produced via single-wavelength exposure to 405 nm, single-wavelength exposure to 532 nm, and dual-wavelength exposure, measured with an applied AC voltage of 100 *V*_pp_ at 1 kHz. (c) (i) POM image of a dual-wavelength exposed 2D periodic binary CLC; (ii) microscopic image of the photomask viewed under POM, with the polarizer (P) and analyzer (A) oriented at 20°, and L indicating the direction of homogeneous alignment.

The meticulous manipulation of LC configurations is essential for advancing high-performance photonic devices. We examined the fabrication of binary CLC cells through two photonic exposure methods: single-wavelength patterned UV exposure and dual-wavelength exposure, which employs patterned UV light in conjunction with uniform green light from the reverse side of the sample. To evaluate the impact of these optical conditions on interfacial boundary sharpness, we utilized 15 μm-thick homogeneously aligned LC cells filled with the specified binary CLC formulation. A photomask depicting the letter “E” defined the spatial characteristics of the UV exposure. Structures were analyzed using polarized optical microscopy (POM), revealing distinct colorimetric responses based on the exposure wavelength. Under short-wavelength illumination, regions treated with azobenzene-based chiral dopants exhibited a yellow-orange hue [41], [42]. In crossed polarizers with a 20° offset, unexposed long-pitch regions appeared orange, while UV-exposed short-pitch domains exhibited a deep red hue. The interface between exposed and unexposed regions demonstrated a high-contrast transition, indicating pitch diffusion across the boundary. Figure 3a(i-ii) demonstrates the gradual enhancement of the gradient observed during 5- and 10-minute UV exposures at 405 nm (0.80 mW/cm^2^). In contrast, Figure 3a(iii-iv) shows the results of simultaneously dual-wavelength exposure using a 532 nm green laser (0.60 mW/cm^2^) applied from behind the sample. Notably, single-wavelength exposure produced broader transition zones characterized by gradual pitch variability ranging from approximately 464 to 532 nm. Conversely, dual-wavelength exposure resulted in sharply defined interfaces. This enhancement is attributed to the spatially modulated isomerization equilibrium of the azobenzene dopants. Under UV-only conditions, *trans*–*cis* isomerization induces a concentration gradient that promotes molecular diffusion. Conversely, applying a green light facilitates the efficient back-isomerization of *cis*-azobenzene to its *trans* form in unexposed regions, effectively minimizing dopant diffusion and preserving spatial fidelity. Furthermore, interfacial boundaries can be sharpened by increasing exposure durations, employing lower-viscosity host materials, or utilizing higher light intensities. These modifications not only expedite diffusion kinetics but also amplify the photochemical back-conversion effects. Our findings underscore the effectiveness of dual-wavelength exposure as a powerful technique for reducing azobenzene diffusion while enhancing the spatial resolution of pitch-modulated chiral nematic LC structures. This paves the way for novel, high-performance photonic applications.

#### 2.2.1 Precision control in dual-wavelength photo-patterning of azo-nCLCs using a 2D photomask

In our pursuit of enhancing spatially periodic photonic structures, we employ a two-dimensional square-patterned photomask with a grid size of 1 mm^2^. This approach enabled precise control over the UV intensity distribution, supplemented by uniform green light exposure from an opposing direction. The experimental setup (Figure S8). Figure 3(b) presents the transmission spectroscopy of the *azo*-*n*CLC cell applied with an AC voltage of 100 *V*_pp_ at 1 kHz, highlighting the distinct spectral responses elicited by various light exposure regimes: UV light, green light, and their combination. The POM images in Figure 3(c-i) illustrate the binary CLCs under dual-wavelength exposure, corresponding to the spectral data in Figure 3(b). Additionally, Figure 3(c-ii) displays the photomask under POM, confirming the periodic grid pattern employed to spatially control UV exposure across the LC cell. The opaque regions of the mask, represented by the orange areas, exhibit reflection spectra ranging from 756 to 821 nm, with a pitch of approximately 506 nm. In contrast, the transparent regions (green areas) yield reflection spectra of about 674–727 nm, corresponding to a pitch near 450 nm. Notably, dual-wavelength exposure resulted in asymmetric reflectance characteristics; the shorter pitch regions (674–727 nm, pitch ∼450 nm) displayed an increasing reflectance trend with wavelength, while the longer pitch regions (756–821 nm, pitch ∼506 nm) exhibited a decreasing reflectance pattern. This spectral asymmetry results from the diffusion of photoresponse azobenzene, where concentration gradients between irradiated and unirradiated areas lead to pitch redistribution from 450 to 506 nm, thus resulting in intermediate reflection bands. To mitigate spectral broadening linked to diffusion, we propose two strategic solutions. The first strategy involves increasing the photomask grid size to restrict lateral diffusion, though this may compromise spatial resolution. The second strategy advocates the implementation of pixelated LC domains that incorporate physical or chemical barriers to confine diffusion to designated regions. This latter approach shows considerable promise for enhancing high-resolution, wavelength-selective optical components.

### 2.3 Spectral filtering using photo-responsive azo-nCLCs for narrowband light source generation

**Figure 4:**
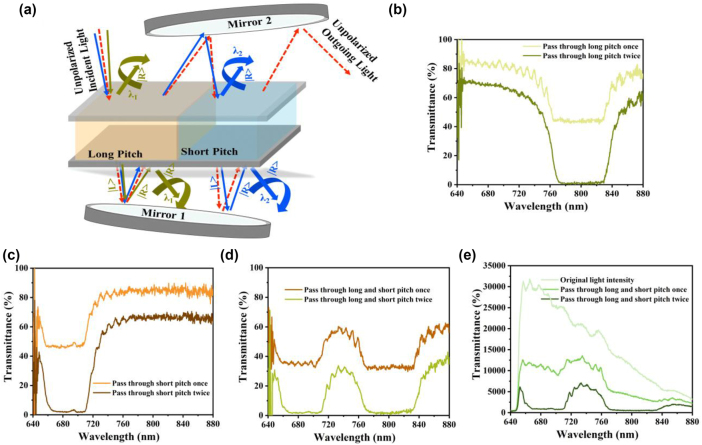
Spectral filtering of reconfigurable narrowband unpolarized light sources. (a) Schematic of spectrum filtering using CLC. (b) Transmission spectrum of the CLC after passing through the long-pitch region of the cell. (c) The transmission spectrum follows traversal through the short-pitch region of the CLC cell. (d) Combined transmission spectrum when light passes through both long- and short-pitch regions of the CLC cell. (e) Comparative analysis of original light (unpolarized) intensity versus intensity after traversing both pitch regions of the CLC cell once and twice.

To establish tunable narrowband light sources for advanced photonic applications, we investigated a spectral filtering approach that utilizes photo-responsive *azo-n*CLC cells. This innovative approach diverges from traditional cholesteric lasers [43] and materials engineering techniques [44], [45], [46], offering an efficient, flexible solution for the selective transmission of specific wavelengths from broadband light sources. Our results unveil a mechanism for precise wavelength control through the intrinsic properties of CLCs, which reflect circularly polarized light within a defined PBG determined by their helical pitch. Upon exposure to UV light at a power density of 5 mW/cm^2^ for 5 min, azobenzene molecules in the CLC undergo *trans*-to-*cis* isomerization, resulting in significant modulation of the helical pitch. This creates two distinct regions within the LC cell: one with a shorter pitch (irradiated area) and another with a longer pitch (nonirradiated area). This localized alteration enables tunable spectral filtering, leveraging multiple light passes through the CLC cell to enhance selectivity, as illustrated in the experimental setup shown in Figure S9. During the filtering process, right-handed circularly polarized light (|R>) is predominantly reflected during its first traversal through the long-pitch region, while left-handed light (|L>) is transmitted. A first mirror (Mirror 1) redirects the transmitted light to invert its handedness, facilitating a second interaction with the CLC. A second mirror (Mirror 2) further directs the light through the short-pitch region, refining the spectral output (Figure 4(a)).

We systematically varied the positioning of the CLC cell within the optical path to evaluate its spectral transmission characteristics under different filtering conditions. Figure 4(b) displays the transmission spectra from light passage through the long-pitch region, both once and twice, and offers a comparison with the short-pitch region (Figure 4(c)). Figure 4(d) shows the spectra observed following sequential light passage through both regions, underscoring the impact of multiple traversals. Specifically, a single pass through the CLC attenuates approximately 50 % of reflected spectral components, while two passes nearly eliminate these components. Spectral elements outside the reflection band exhibited minimal intensity loss, attributable to cumulative reflections and scattering at material interfaces. In Figure 4(e), we compare the initial broadband intensity with the outputs after one and two filtering cycles, demonstrating effective suppression within the targeted band while preserving broadband characteristics beyond 850 nm. Notably, the light filtered outside the reflection band remains unpolarized, indicating negligible distortion during the filtering process. This unpolarized filtering effect is achieved through sequential interactions of light with regions of short and long pitch situated between two mirrors, paralleling the principles of Fabry–Perot unpolarized filtering mechanisms [47]. Our dual-wavelength patterned CLC architecture presents a passive, tunable, and spatially programmable solution, eliminating the need for additional polarization management. Future optimization may involve fine-tuning the position and width of the reflection band through variations in dopant concentration or the application of dual-wavelength photoirradiation (UV and green), thereby enhancing dynamic modulation of adjacent Bragg bands. Overall, this technique constitutes a reconfigurable, passive mechanism for generating narrowband unpolarized light, highlighting its significant potential for integration into compact, wavelength-selective photonic devices and sensors.

## 3 Conclusions

In summary, we present a pioneering dual-wavelength photoisomerization method that allows for precise manipulation of the spectral sculpting properties of photonic materials using *azo-n*CLCs. The prepared device enables reversible *trans*–*cis* isomerization of chiral dopants, thereby enabling dynamic adjustment of the helical pitch, an essential parameter for spectral tuning through concurrent excitation at 405 nm and 532 nm. We have achieved impressive bidirectional shifts in the reflection band, exceeding 140 nm in spectral range and 100 nm in tunable bandwidth. By optimizing beam intensity ratios and adjusting propagation geometries, we can tailor customized pitch domains to significantly alter the reflection bandwidth and central wavelength, achieving remarkable spectral and spatial control. Our counterpropagating beam configurations offer smooth pitch gradients, enabling continuous tuning that surpasses traditional copropagating methods. Additionally, employing photomasking with dual-beam irradiation enables the creation of sharply defined pitch domains with minimal molecular diffusion, which is essential for high-resolution binary and pixelated CLC architectures. This passive operation requires no electrical bias and supports reconfigurable optical filtering and spectral shaping. The inherent circular polarization selectivity of our CLCs promotes narrowband reflection and dynamic light routing, establishing a versatile framework for light-programmable systems. Notably, our multipass optical architecture improves spectral discrimination through polarization-selective reflection, effectively filtering undesired spectral components with high fidelity. Overall, this groundbreaking approach to isomerization-driven photonic bandgap engineering enables spatially programmable, multiband reflections with sub-nanometer precision, opening new avenues for energy-efficient optics and integrated optical communication systems.

## 4 Experimental section/methods

### 4.1 Preparation of the *azo-n*CLCs cell

CLC mixtures were prepared by adding a right-handed chiral dopant (R1011, HTP ≈ +32 μm^−1^) along with a photo-responsive azobenzene-based chiral dopant (ChAD-2-S, BEAM Co.) into a nematic host that exhibits negative dielectric anisotropy (HNG30400-200, FUSOL MATERIAL CO., LTD). The CLC mixture was carefully formulated with a weight ratio of 88.8:7.3:3.9 for the nematic host, chiral azobenzene, and chiral host, respectively. The unique azobenzene dopant undergoes wavelength-dependent isomerization between its *trans* state (HTP ≈ −12.4 μm^−1^) and *cis* state (HTP ≈ 0 μm^−1^ due to the doped chiral dopant, R1011), allowing for dynamic modulation of CLC properties. The mixture was confined between two glass substrates, each treated with an indium tin oxide coating and a unidirectionally rubbed polyvinyl alcohol alignment layer to ensure uniform planar alignment. To construct the cell, the glass substrates were arranged with 30 μm-thick spacers and filled with the CLC mixture using a capillary action technique. This carefully engineered platform enables tunable modulation of the helical pitch via photoinduced *trans*–*cis* isomerization of ChAD-2-S, achieved through selective laser exposure at 405 nm and 532 nm. This innovative capability facilitates dynamic and reversible control over the spectral characteristics of the Bragg reflection band within the CLC, representing a significant advancement toward the development of reconfigurable photonic applications.

### 4.2 Measurement setup

To examine the *azo-n*CLCs Cell, we employed a 405 nm and 532 nm Nd: YAG laser (Unice E-O Services Inc.). A DET36A photodetector (Thorlabs) and a DS4034 digital oscilloscope (RIGOL Technologies) were used to facilitate detection and analysis. A standard tungsten halogen lamp (HL-2000, Ocean Optics, Inc.) served as the light source, with intensity controlled by an attenuator to keep levels within the saturation limits of the spectrometer. To prevent the short-wavelength component of the probe light from affecting the azobenzene material in the LC mixture, a 650 nm high-pass filter was installed prior to the probe light entering the *azo-n*CLCs Cell.

## Supplementary Material

Supplementary Material Details
