# Supplementary material for: Light-guided spectral sculpting in chiral azobenzene-doped cholesteric liquid crystals for reconfigurable narrowband unpolarized light sources
Source: Nanophotonics. 2025 Dec 4;14(27):5395–405. doi: 10.1515/nanoph-2025-0455 (PMC12717891; doi:10.1515/nanoph-2025-0455)
Supplement: Supplementary file 1 — Supplementary Material Details [file j_nanoph-2025-0455_suppl_001.docx]

Supporting Information

**Light-Guided Spectral Sculpting in Chiral Azobenzene-Doped Cholesteric Liquid Crystals for Reconfigurable Narrowband Unpolarized Light Sources**

Pravinraj Selvaraj ^†^, Ming-Hong Yuan ^†^, Cheng-Kai Liu, and Ko-Ting Cheng*

*Department of Optics and Photonics, National Central University, Taoyuan City 320317, Taiwan*

^†^These authors contributed equally.

***[*chengkt@dop.ncu.edu.tw*](mailto:chengkt@dop.ncu.edu.tw)


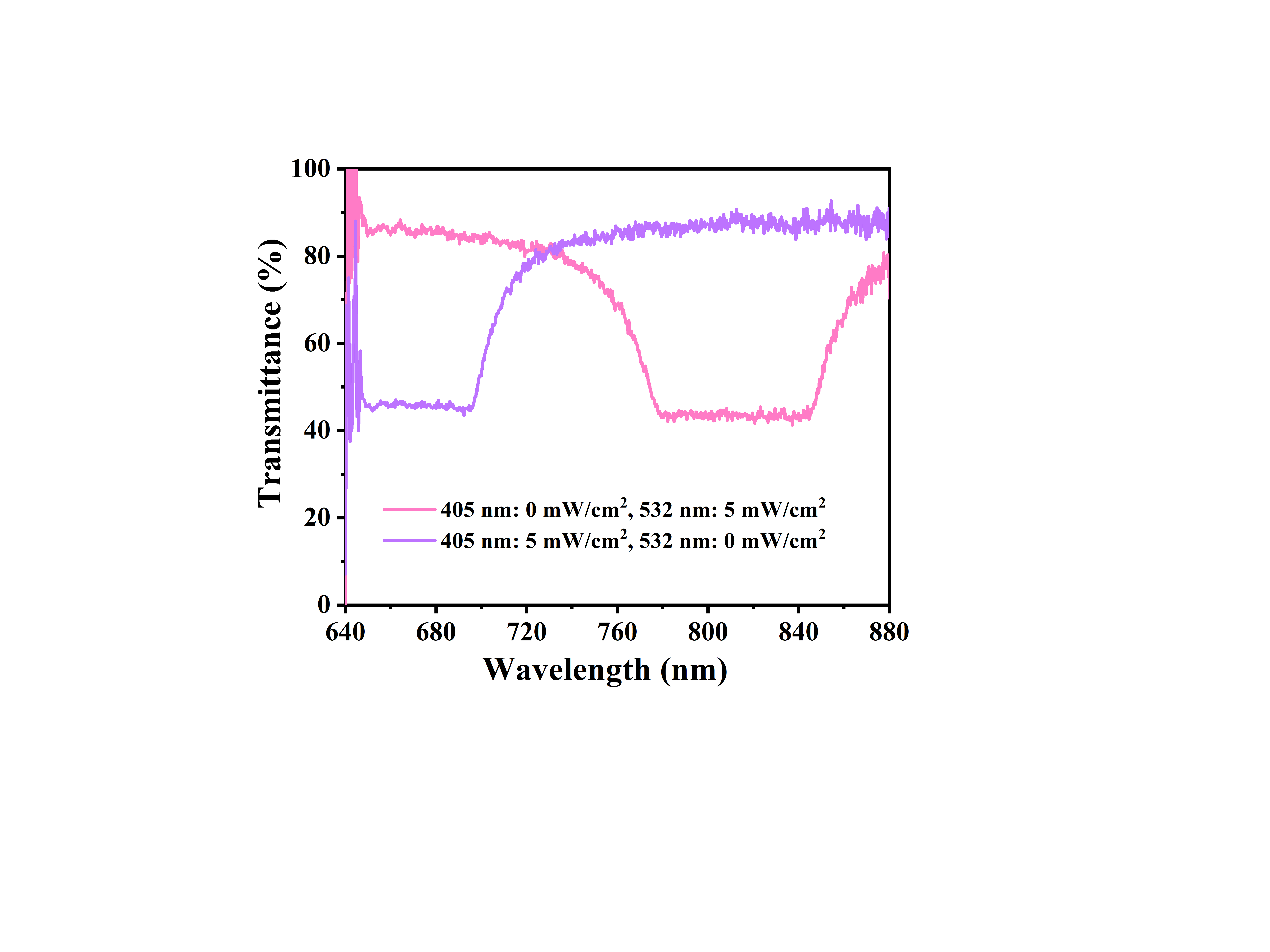


**Figure S1.** Transmission spectra of the *azo-n*CLC cell under counter-propagation at varying wavelengths: the *trans*-isomer (532 nm) and the *cis*-isomer (405 nm), measured with an AC voltage of 100 V_pp_ at 1 kHz.


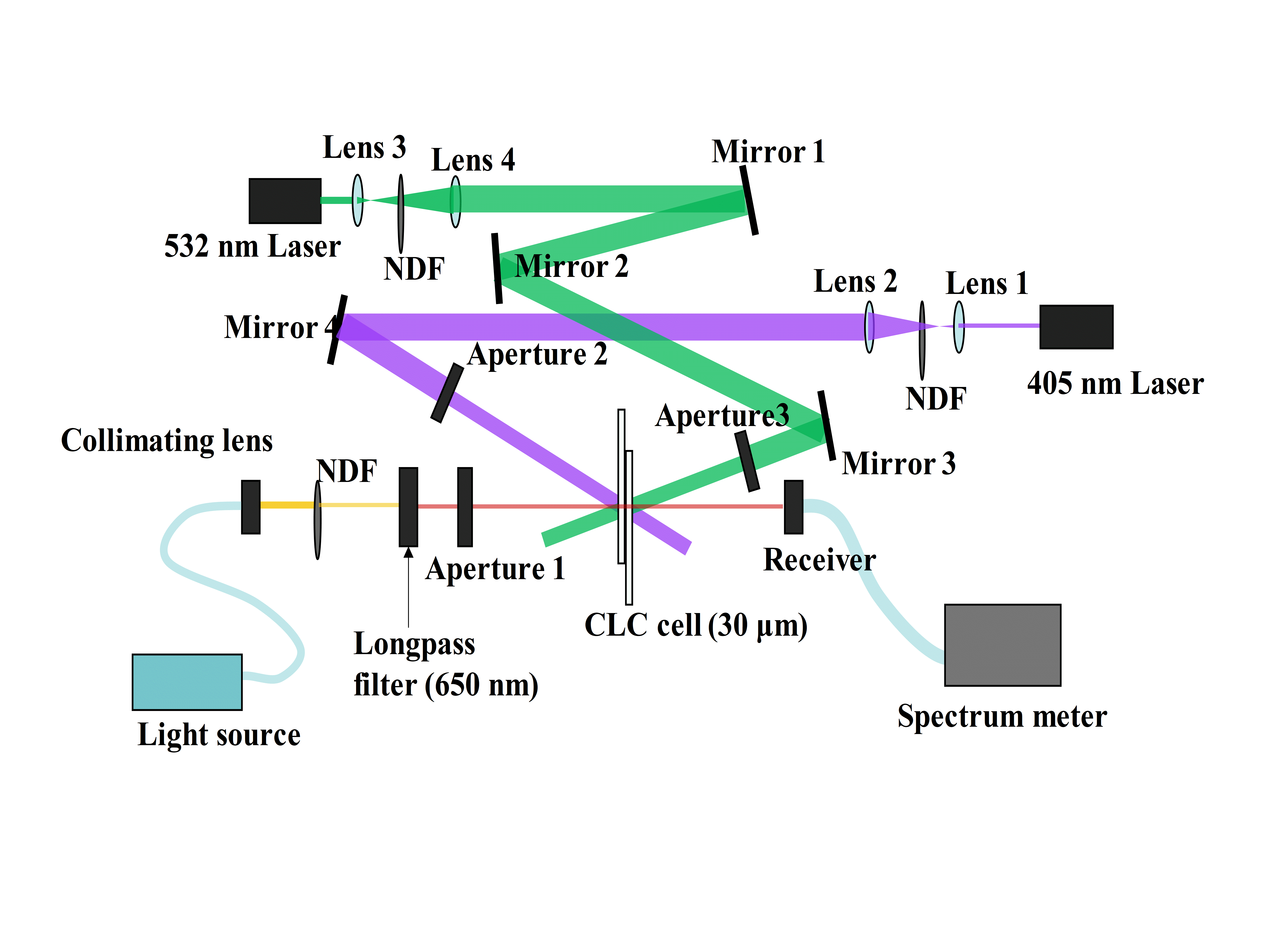


**Figure S2.** Schematic illustrating the optical path employed to modulate the reflection spectrum of CLCs using counter-propagation dual-wavelength exposure.


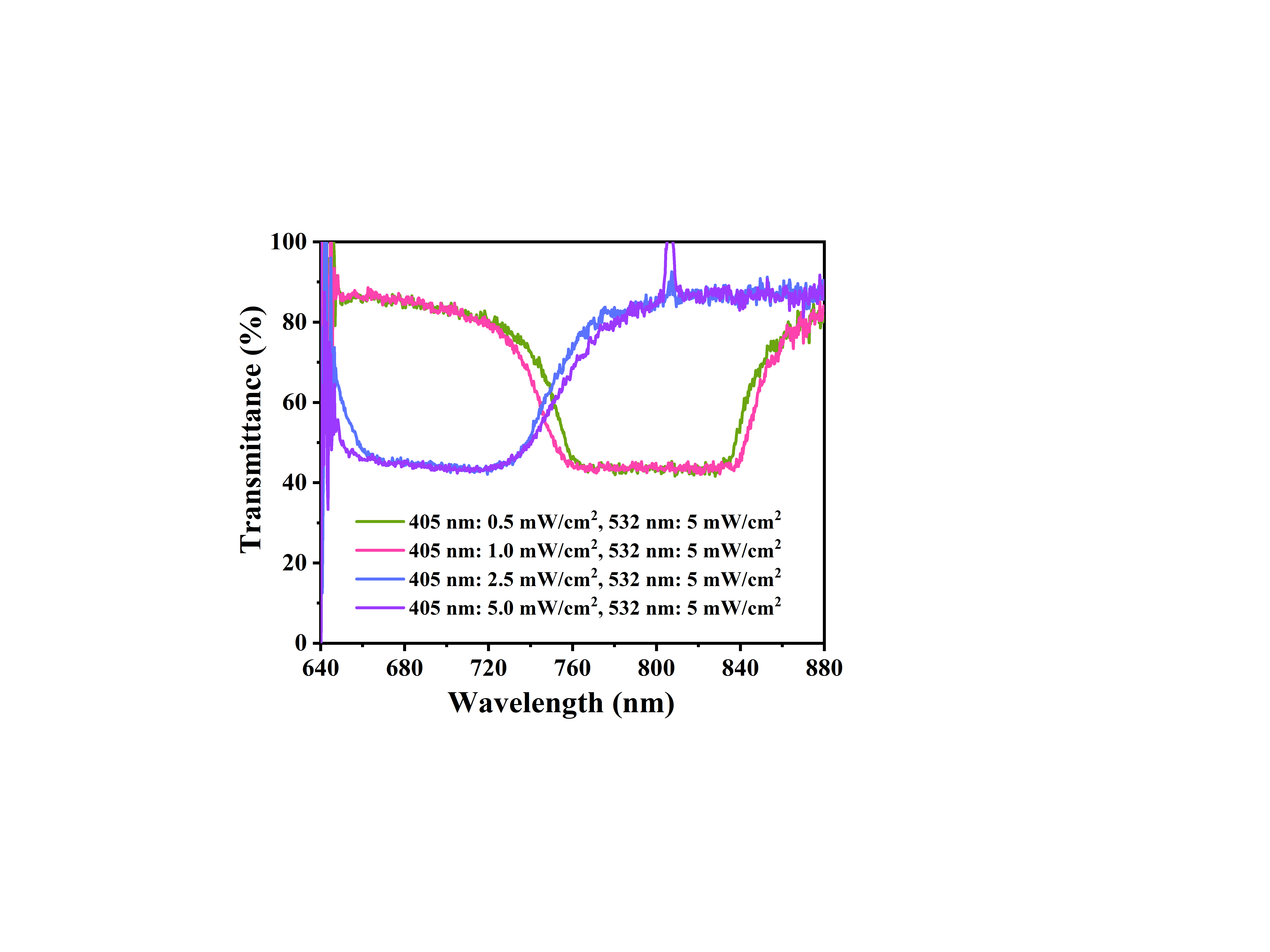


**Figure S3:** Transmission spectra of CLCs under UV-to-green intensity ratios of 20:1 and 1:1, measured with an AC voltage of 100 V_pp_ at 1 kHz.


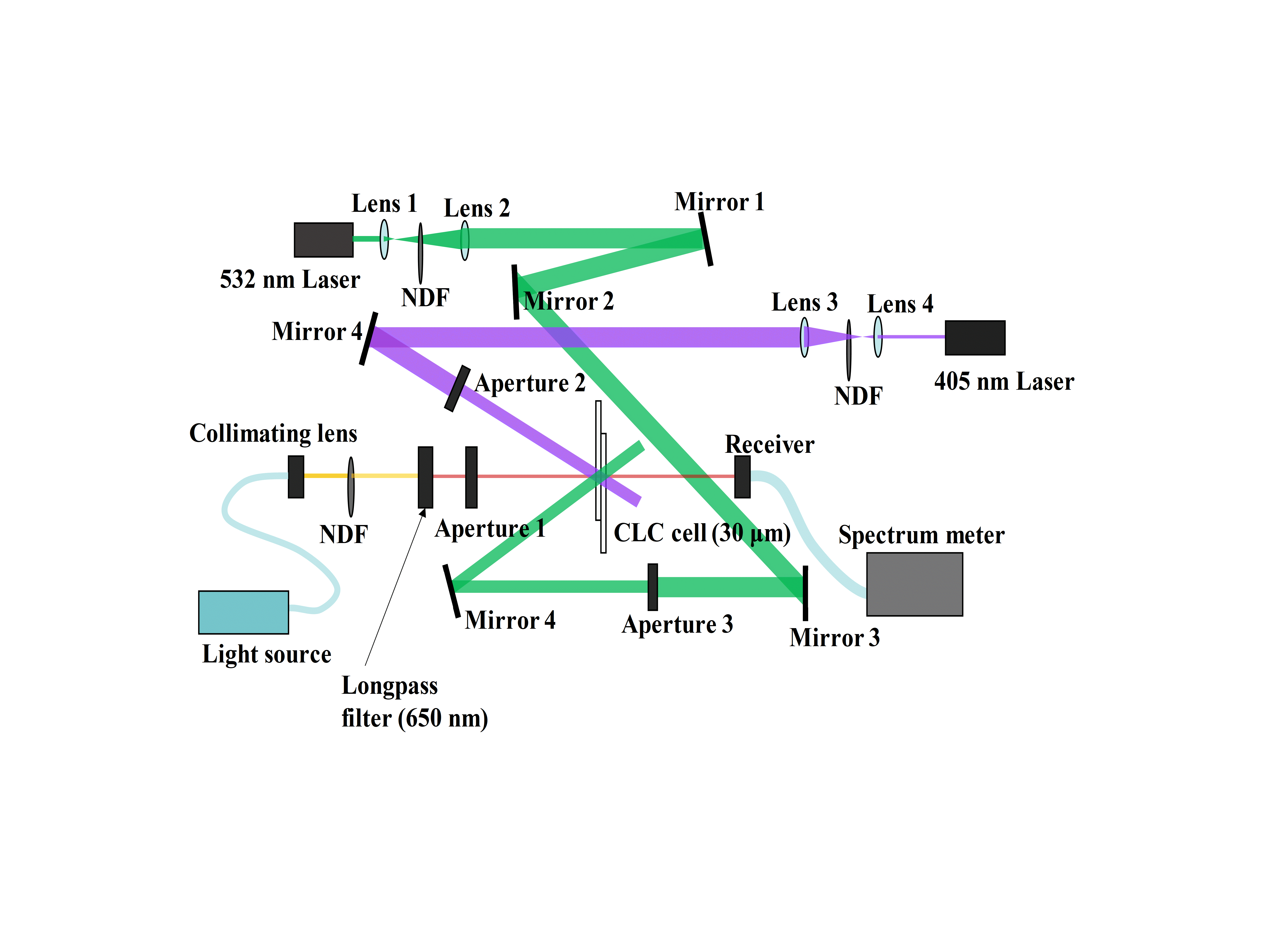


**Figure S4.** Schematic illustration of the optical path employed to modulate the reflection spectrum of CLCs through co-propagation dual-wavelength exposure.


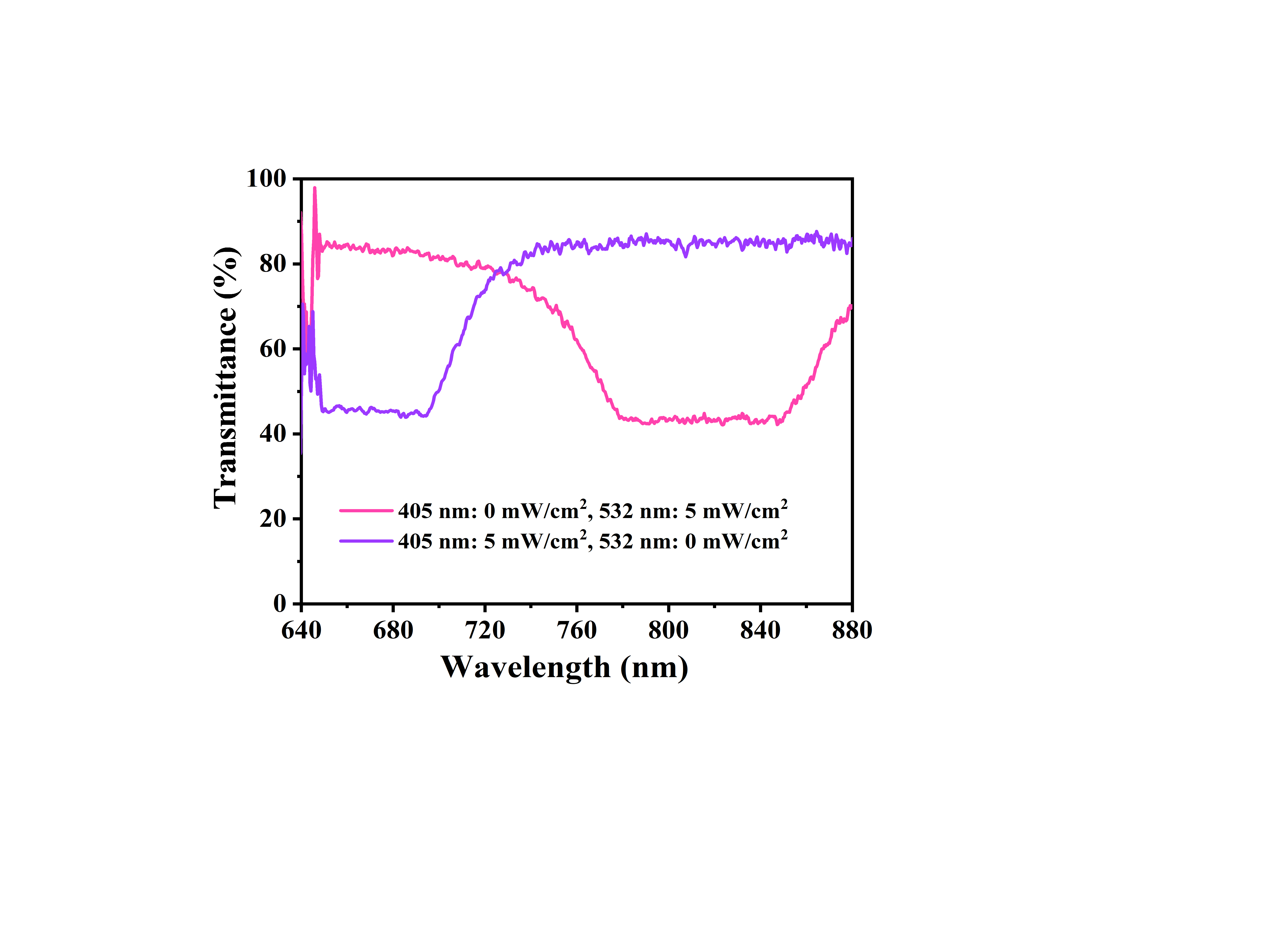


**Figure S5.** Transmission spectra for the ChAD-2-S-doped CLC cell under co-propagation at specific wavelengths corresponding to the *trans* isomer (532 nm) and the *cis* isomer (405 nm), with an AC voltage of 100 V_pp_ at 1 kHz.


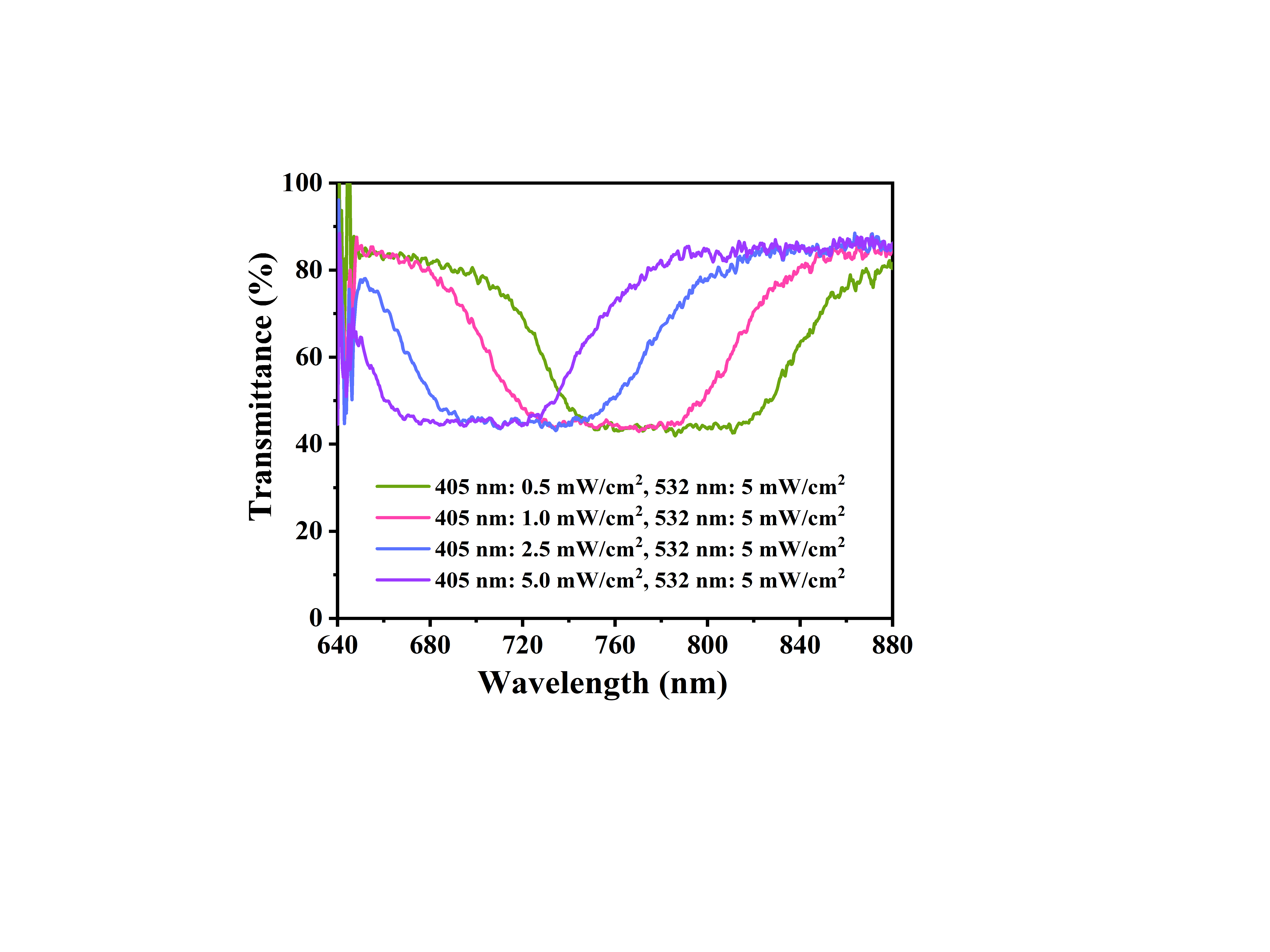


**Figure S6.** Transmission spectra of a ChAD-2-S-doped CLC cell subjected to simultaneous dual-wavelength exposure with a fixed 5.0 mW/cm² 532 nm laser and a 405 nm laser of varying intensities with an AC voltage of 100 V_pp_ at 1 kHz.


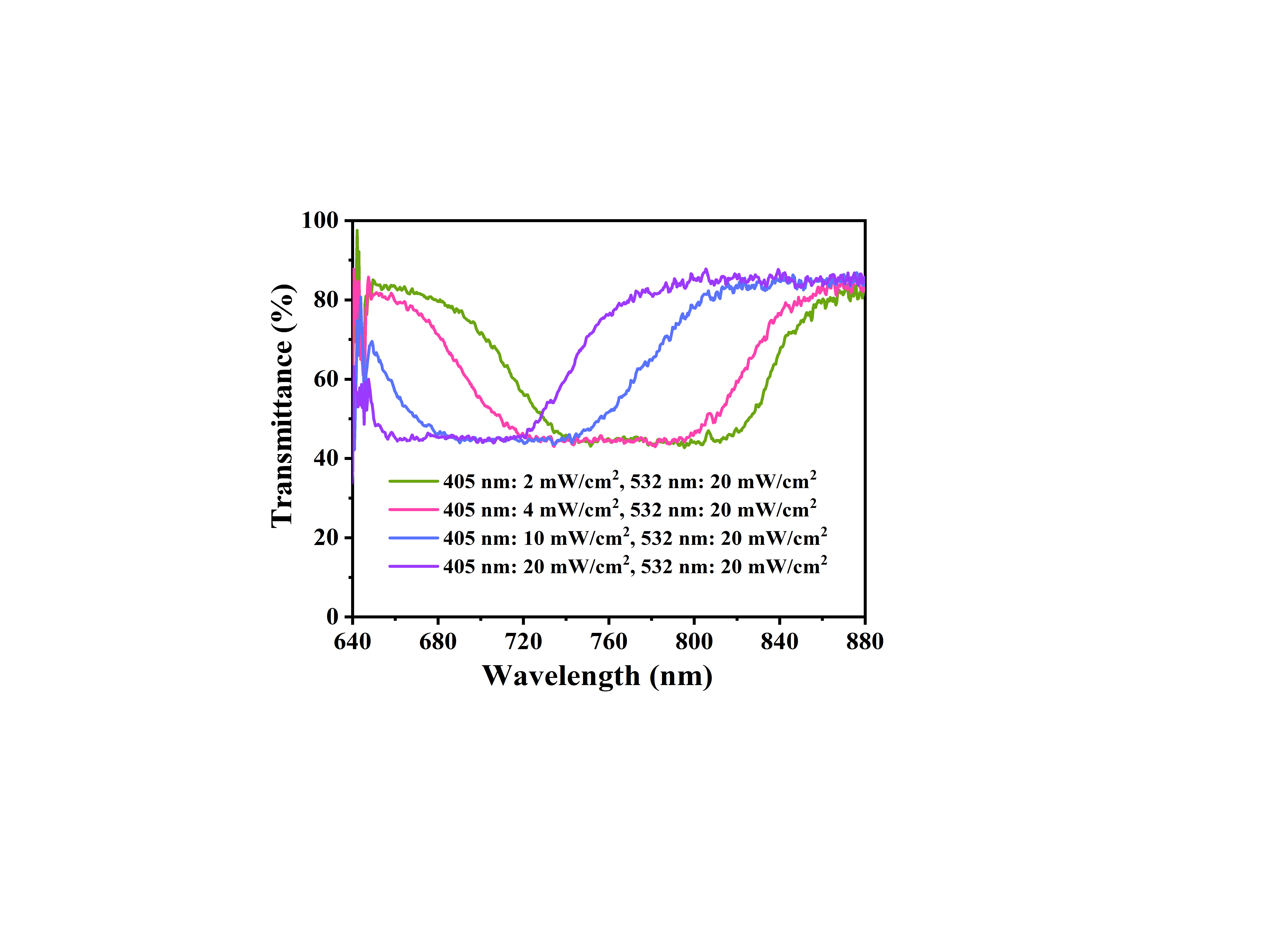


**Figure S7.** Transmission spectra of a ChAD-2-S-doped CLC cell under simultaneous dual-wavelength exposure to a fixed 20 mW/cm² 532 nm laser and varying intensities of a 405 nm laser with an AC voltage of 100 V_pp_ at 1 kHz.


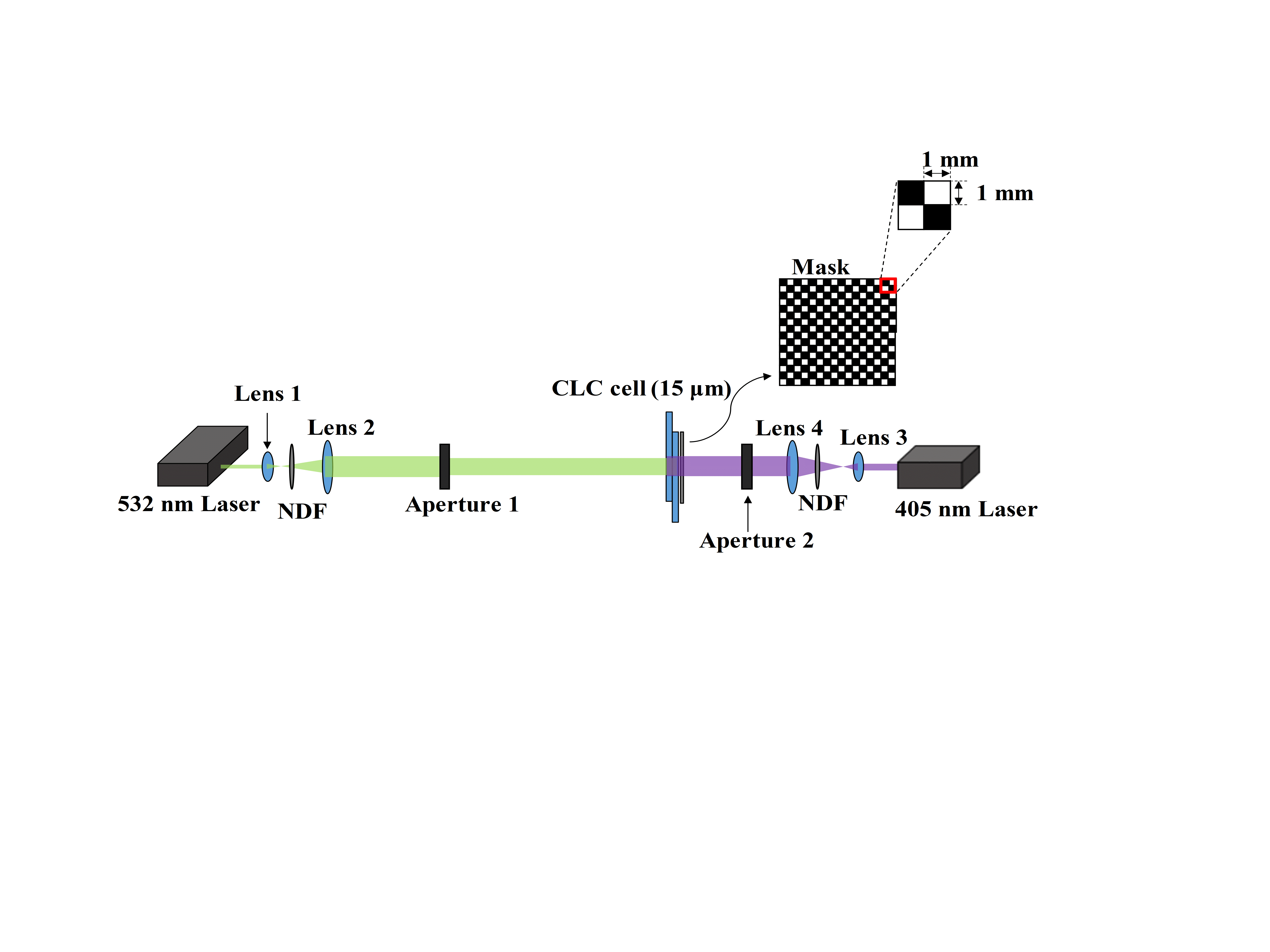


**Figure S8.** Schematic illustration of the optical setup employed for the fabrication of spatially modulated binary CLCs using counter-propagation dual-wavelength exposure and a 2D-periodic photomask.

**
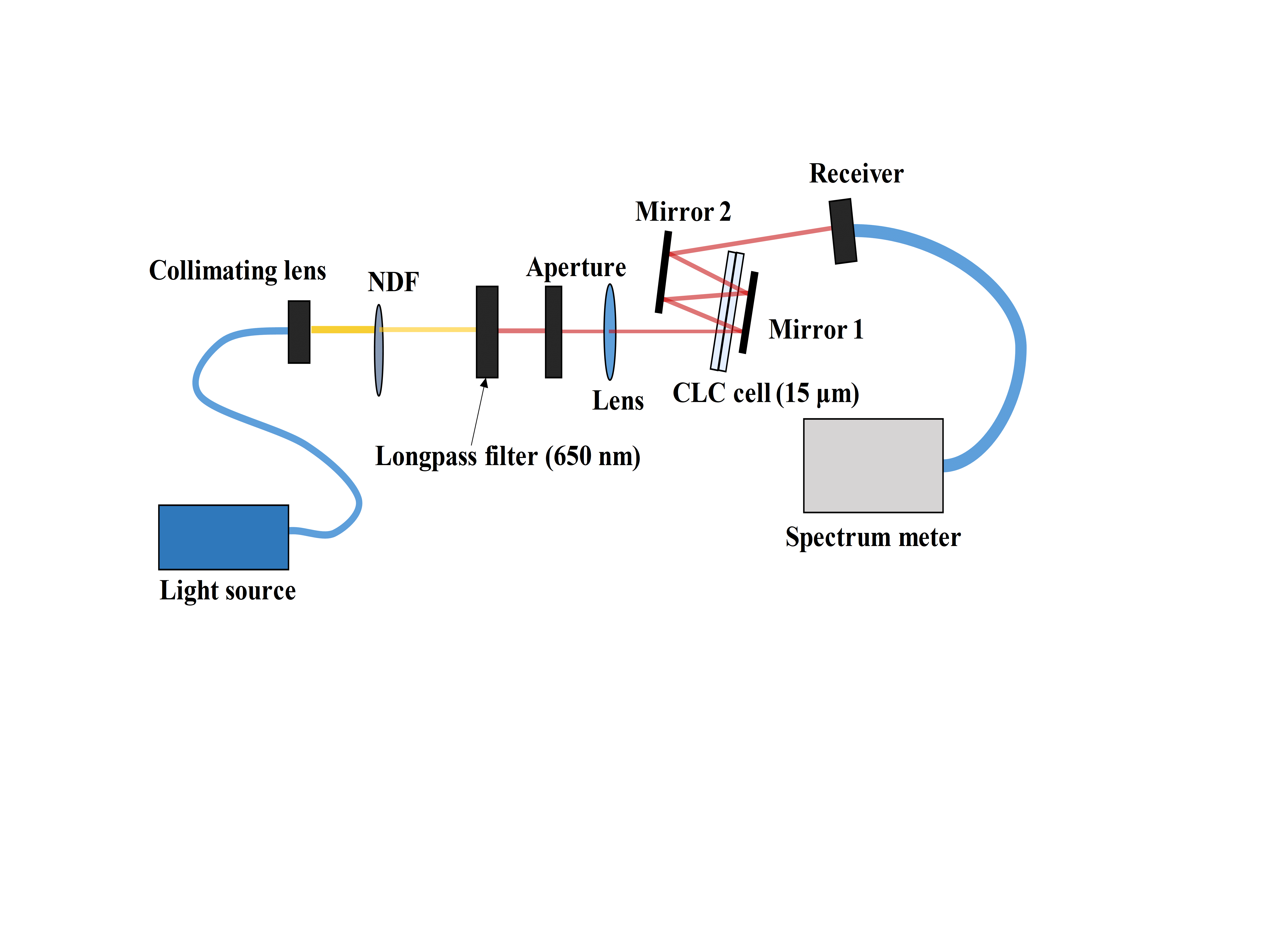
**

**Figure S9.** Schematic illustration of the optical setup employed for generating narrowband light.

**Table 1.** Comparison of central wavelengths and bandwidths of the reflection spectrum under varying UV and green light intensities, in counter-propagating and co-propagating configurations.

| **Intensity (mW/cm^2^)**  405 nm / 532 nm | **Counter-propagation** | | **Co-propagation** | |
| --- | --- | --- | --- | --- |
|  | $\bar{\text{λ}}$ (nm) | Δλ (nm) | $\bar{\text{λ}}$ (nm) | Δλ (nm) |
| **0 / 5.0** | 811 | 73 | 815 | 78 |
| **5 / 0** | 665 | 60 | 666 | 63 |

**Table 2.** Comparison of the reflected spectrum's central wavelengths and bandwidths at varying UV light intensities, with a fixed green light intensity of 5.0 mW/cm², in counter-propagating and co-propagating configurations.

| **Intensity (mW/cm^2^)**  405 nm / 532 nm | **Counter-propagation** | | **Co-propagation** | |
| --- | --- | --- | --- | --- |
|  | $\bar{\text{λ}}$ (nm) | Δλ (nm) | $\bar{\text{λ}}$ (nm) | Δλ (nm) |
| **0.5 / 5.0** | 785 | 79 | 782 | 75 |
| **1.0 / 5.0** | 762 | 81 | 757 | 70 |
| **2.5 / 5.0** | 725 | 77 | 721 | 66 |
| **5.0 / 5.0** | 698 | 73 | 697 | 65 |

**Table 3.** Comparison of the reflected spectra central wavelengths and bandwidths at varying UV light intensities, with a fixed green light intensity of 20.0 mW/cm², in counter-propagating and co-propagating configurations.

| **Intensity (mW/cm^2^)**  405 nm / 532 nm | **Counter-propagation** | | **Intensity (mW/cm^2^)**  405 nm / 532 nm | **Co-propagation** | |
| --- | --- | --- | --- | --- | --- |
|  | $\bar{\text{λ}}$ (nm) | Δλ (nm) |  | $\bar{\text{λ}}$(nm) | Δλ (nm) |
| **0 /20.0** | 812 | 72 | **2.0 / 20.0** | 78 | 88 |
| **2.5 / 20.0** | 778 | 101 | **4.0 / 20.0** | 57 | 87 |
| **5.0 / 20.0** | 754 | 109 | **10.0 / 20.0** | 15 | 75 |
| **20.0 / 20.0** | 694 | 83 | **20.0 / 20.0** | 89 | 73 |

**Table 4.** Comparison of the central wavelengths and bandwidths of the reflected spectrum at varying intensities of UV light and green light, in counter-propagating configurations.

| **Light intensity (mW/cm^2^)**  405 nm / 532 nm | **Counter-propagation** | | |
| --- | --- | --- | --- |
|  | $\bar{\text{λ}}$(nm) | Δλ (nm) | |
| 0.25 / 5.0 | 797 | | 78 |
| 1.0 / 20.0 | 797 | | 87 |
| 5.0 / 5.0 | 698 | | 72 |
| 20.0 / 20.0 | 694 | | 82 |
